# Effects of Changes in Adiposity and Physical Activity on Preadolescent Insulin Resistance: The Australian LOOK Longitudinal Study

**DOI:** 10.1371/journal.pone.0047438

**Published:** 2012-10-12

**Authors:** Richard D. Telford, Ross B. Cunningham, Rohan M. Telford, Jennifer Kerrigan, Peter E. Hickman, Julia M. Potter, Walter P. Abhayaratna

**Affiliations:** 1 College of Medicine, Biology and Environment, Australian National University, Canberra, Australia; 2 Clinical Trials Unit, Canberra Hospital, Canberra, Australia; 3 Fenner School of Environment and Society, Australian National University, Canberra, Australia; 4 Centre for Research and Action in Public Health, Faculty of Health, University of Canberra, Canberra, Australia; 5 ACT Pathology, Canberra Hospital, Canberra, Australia; John Hopkins Bloomberg School of Public Health, United States of America

## Abstract

**Background:**

In a previous longitudinal analysis of our cohort as 8 to 10 year-olds, insulin resistance (IR) increased with age, but was not modified by changes in percent body fat (%BF), and was only responsive to changes in physical activity (PA) in boys. We aimed to determine whether these responses persisted as the children approached adolescence.

**Methods:**

In this prospective cohort study, 256 boys and 278 girls were assessed at ages 8, 10 and 12 years for fasting blood glucose and insulin, %BF (dual energy X-ray absorptiometry); PA (7-day pedometers), fitness (multistage run); and pubertal development (Tanner stage).

**Results:**

From age 8 to 12 years, the median homeostatic model of IR (HOMA-IR) doubled in boys and increased 250% in girls. By age 12, 23% of boys and 31% of girls had elevated IR, as indicated by HOMA-IR greater than 3. Longitudinal relationships, with important adjustments for covariates body weight, PA, %BF, Tanner score and socioeconomic status showed that, on average, for every 1 unit reduction of %BF, HOMA-IR was lowered by 2.2% (95% CI 0.04–4) in girls and 1.6% (95% CI 0–3.2) in boys. Furthermore, in boys but not girls, HOMA-IR was decreased by 3.5% (95%CI 0.5–6.5) if PA was increased by 2100 steps/day.

**Conclusion:**

Evidence that a quarter of our apparently healthy 12 year-old Australians possessed elevated IR suggests that community-based education and prevention strategies may be warranted. Responsiveness of IR to changes in %BF in both sexes during late preadolescence and to changes in PA in the boys provides a specific basis for targeting elevated IR. That body weight was a strong covariate of IR, independent of %BF, points to the importance of adjusting for weight in correctly assessing these relationships in growing children.

## Introduction

Physical activity (PA), adiposity and cardiorespiratory fitness (CRF) are lifestyle-related factors known to influence insulin resistance (IR) and the risk of type 2 diabetes mellitus in adults [1]. Cross-sectional studies of these relationships in children have been well demonstrated [2] but there have been limited data acquired longitudinally to evaluate serial changes within a child. Longitudinal relationships provide a higher level of evidence toward causality compared with cross-sectional relationships as they are less affected by genetic and environmental (e.g. family related) confounders. A previous publication involving this cohort of children between 8 and 10 years of age [3] revealed no evidence of any longitudinal relationships between IR and percent body fat (%BF) in boys or girls, and evidence of a longitudinal relationship between IR and PA occurred only in the boys.

We aimed to extend the mid-study findings by analyzing data in the same cohort through to 12 years of age to provide a more complete picture of the progression of preadolescent IR and its responsiveness to changes in %BF and PA. In particular we set out to determine whether the lack of effect of %BF on IR, and the gender differences in the influence of PA persisted as the children approached puberty. It was also of interest to quantify the changes in %BF and PA required to make practically significant impacts on IR.

Furthermore, given that we have a representative sample of community-based children and that, based on secular trends, 25% of these children are likely to develop disorders of glucose metabolism and type 2 diabetes as adults [4], we aimed to determine the prevalence of elevated IR in a cohort of apparently healthy Australian children using the suggested cutoff point for the homeostatic model for insulin resistance (HOMA-IR) of 3 [5]. It was anticipated that these data, together with gender specific effects of PA and body composition changes might then assist making recommendations for any future strategies directed at moderating IR during pre-adolescence.

## Materials and Methods

In this prospective cohort study, children were recruited from 29 government-funded elementary (primary) schools situated in outer suburbs of a city of population approximately 330,000. The suburbs in which the schools were situated were relatively homogeneous in terms of socioeconomic status, data from the Australian Bureau of Statistics indicating average family income of each suburb to be close to the Australian average at the time of recruitment [6]. This study was part of the multidisciplinary Lifestyle of our Kids (LOOK) project, the breadth of which has been previously described [7]. In short, the LOOK study has two major objectives; firstly to investigate the effect of changes in PA and %BF on a range of physiological and psychological characteristics during preadolescence, the current paper reporting on IR. Secondly, it was to investigate the effect of a specialist physical education program during the final four years of elementary (primary) school, its effect on IR to be described in a separate paper. A condition of inclusion in the study was that children were in good health and able to participate freely in vigorous physical activity. Approximately 90% of the children had White parents; 8% were of Asian descent; 1% of Indigenous Australian or Polynesian descent, and we had no data on the ethnicity of 1% of the families.

Parents or guardians provided informed written consent for the children to undergo evaluations during three main measurement periods, at ages 8, 10 and 12 years of age at the end of grades 2, 4, and 6 of elementary school. Body composition was measured in a hospital setting, using DXA (dual energy X-ray absorptiometry, Hologic Discovery QDR Series, Hologic Inc., Bedford, MA, USA (DXA HD)). All scans were performed with children wearing light clothing and total body scans were analyzed using QDR Hologic Software Version 12.4.7 to generate total lean tissue mass and fat mass from which %BF was calculated. Height was measured by a portable stadiometer to the nearest 0.001 m and body weight by portable electronic scales to the nearest 0.05 kg. To facilitate comparisons of this cohort with other populations we measured body mass index (BMI) and classified the children according to the published and widely used values depicting overweight and obesity [8].

A 20-meter multistage shuttle test was used to estimate CRF, being well-established as a field test with children [9]. In the assessment of PA, the children wore pedometers on their hip for seven consecutive days, the first day's measurements being ignored on the premise that the novelty of wearing the pedometers may influence the initial activity. The pedometers were Walk 4 Life (Plainfield, IL USA) sealed pedometers, recommended for use with children [10]. A PA index was calculated as previously reported [11] to meet linearity assumptions of our statistical model and to account for missing values, and is approximately the square root of the average number of steps per day.

Fasting blood samples were collected by nurses experienced in pediatric phlebotomy in the school setting before school, breakfast being supplied following the procedure. Children were given specific instructions and reminders to fast, and all children and their parents, when present, were questioned in this regard to ensure this took place. Fasting blood samples were collected in the school setting and serum samples were mixed and allowed to clot for up to 30 minutes prior to centrifugation. Samples were centrifuged for 10 minutes at 2850 rpm (Spintron GT-25P, Spintron Pty Ltd, Australia) and either immediately frozen in dry ice and stored at −80°C for subsequent analysis or taken to ACT Pathology at Canberra Hospital for immediate analysis. Serum glucose concentration was measured by hexokinase colorimetric methodology on the Architect Ci8200 (Abbott laboratories, IL 60064 USA). Serum insulin concentration was measured using microparticle enzyme immunoassay on the AXSYM (Abbott laboratories, IL 60064 USA). Our marker of choice was the homeostatic model of IR, HOMA-IR [12], where HOMA-IR is fasting insulin (mU/L)×fasting glucose (mmol/L) all divided by 22.5. Whilst insulin sensitivity may be a more appropriate term in asymptomatic children, HOMA-IR has been validated for use with children [13], [14] and can be compared with previous publications in children [15], [16], [17].

Pubertal development was determined at ages 10 and 12 years, each child carrying out a self-assessment of Tanner stages [18] using diagrams based on those previously described [19]. This was completed in the privacy of their homes with parental supervision at age 10 years, and in the hospital setting at age 12 years under the supervision of a qualified teacher who was well known to the children.

### Statistical analysis

A detailed description of the statistical analysis is supplied as Statistics S1. Summarizing the main features, we used a model that fits within the general framework of general linear mixed models [20], and which was designed to quantify relationships between the response variable IR and explanatory variables PA, weight, %BF, CRF, socioeconomic status, and puberty assessments. The model allowed us to segregate overall relationships into the cross-sectional (between-child) and the longitudinal (within-child) levels. Furthermore, as indicated in the appendix, given that half of these children undertook an intervention of specialized physical education at school while the other half continued with their normal physical education, the model included an adjustment factor in the unlikely event that relationships between IR and the PA or %BF were affected by the intervention.

This study was approved by the human research ethics committees representing both the Australian Capital Territory Department of Health and the Australian Institute of Sport.

## Results

### Participant characteristics and Attrition

Characteristics of the study participants are detailed in Table 1. The numbers of observations varied with the test and the year. There were 715, 562 and 469 blood collections at the three measurement periods, respectively; with similar numbers for each of %BF, PA and CRF. All data were used to quantify the levels of IR in this population. For the longitudinal relationships, the general linear mixed methods model was designed to avoid discarding data where possible, and adjusts for any missed measurement. Twenty children withdrew from the study, and the remaining missed tests were due either to relocations to schools outside the study (n = 165), absences from school on test days (n = 40), inadequate compliance to test procedures including failure to fast and unable to make another appointment or to technical difficulties with the blood collections (n = 21). Children who missed one or more assessments in a particular year remained in the study and were included in the analysis, the statistical model adjusting for missing values. An analysis of baseline data from children who left the study in grade 4 or grade 6 revealed no evidence of any differences in the means of body weight, %BF or PA index with those of children remaining in the study for all three periods (all p>0.3). The respective means and standard errors at baseline of children remaining in the study and those dropping out were: body weight (kg) of boys 28.8 (0.36) v 29.0 (0.41) and of girls 28.4 (0.38) v 28.9 (0.41); %BF of boys 22.6 (0.40) v 22.7 (0.53) and of girls 27.6 (0.42) v 28.5 (0.50); PA index (square root of average steps per day) of boys 108.1 (0.69) v 108.1 (0.77) and of girls 97.9 (0.73) v 96.5 (0.78).

**Table 1:** Unadjusted median values, with 5^th^ and 95^th^ percentiles, with numbers of observations of anthropometry, physical activity, fitness, percent body fat and pubertal maturation stage classified by elementary school grade, gender and expressed as medians and percentiles.

		Grade 2, age 8.1±0.4 y						Grade 4, age 10.1±0.3 y				Grade 6, age 12.1±0.4 y		
		5%	Med			95%		5%	Med	95%		5%	Med	95%
		N = 365 F, 369 M						N = 290 F, 296 M				N = 256 F, 278 M		
Ht	F	120.1			128.6	137.3		130.3	140.5		150.3	141.5	154.1	164.7
cm	M	120.6			130.2	139.4		130.9	141.9		152.0	141.2	153.5	166.3
Wt	F	21.7			27.3	39.8		26.4	34.5		53.0	32.7	45.00	66.2
kg	M	22.5			28.0	38.6		27.2	35.5		50.3	33.2	44.75	66.5
BMI	F	14.1			16.6	22.4		14.5	17.7		24.7	15.2	19.0	26.2
kg/m^2^	M	14.2			16.5	21.1		14.7	17.6		23.7	15.5	18.9	25.3
PA I^1^	F	80.1			97.3	113.4		78.8	93.5		110.7	77.1	91.4	106.5
**√**steps/d	M	90.4			107.9	126.9		84.5	102.2		120.1	78.5	98.0	116.5
CRF	F	2.2			3.2	5.4		2.6	4.1		7.3	3.0	5.2	8.9
run stage	M	2.2			4.1	6.9		2.8	5.4		8.7	2.9	6.2	10.1
%BF	F	19.2			27.1	39.5		18.5	28.5		41.9	18.3	26.6	39.5
	M	15.3			21.8	34.3		15.6	23.9		36.7	14.2	23.5	38.6
Tanner stage	F	not assessed						1.0	1.5		2.6	1.5	2.5	4.0
	M							1.0	1.5		2.5	1.0	2.5	4.0
Glucose	F	4.0		4.6			5.4	4.4	5.0		5.7	4.7	5.3	5.9
mmol/L	M	4.0		4.7			5.6	4.5	5.2		5.7	4.8	5.3	5.9
Insulin	F	2.4		5.0			11.8	3.1	7.4		16.1	5.4	11.3	24.1
IU/L	M	1.9		4.6			9.80	2.7	5.5		13.9	3.9	7.5	16.6
HOMA-IR	F	0.4		1.0			2.6	0.6	1.6		4.0	1.2	2.6	6.4
	M	0.3		0.9			2.1	0.5	1.2		3.3	0.8	1.7	4.3

1 PAI is the physical activity index, approximately equal to the square root of average daily steps per day. CRF is cardiorespiratory fitness, the number of stages reached in the multistage run.

%BF is percentage of body fat as determined by DXA scan. Tanner stage is the self-assessed pubertal stage ranking.

### Changes in Insulin Resistance

**Figure 1:**
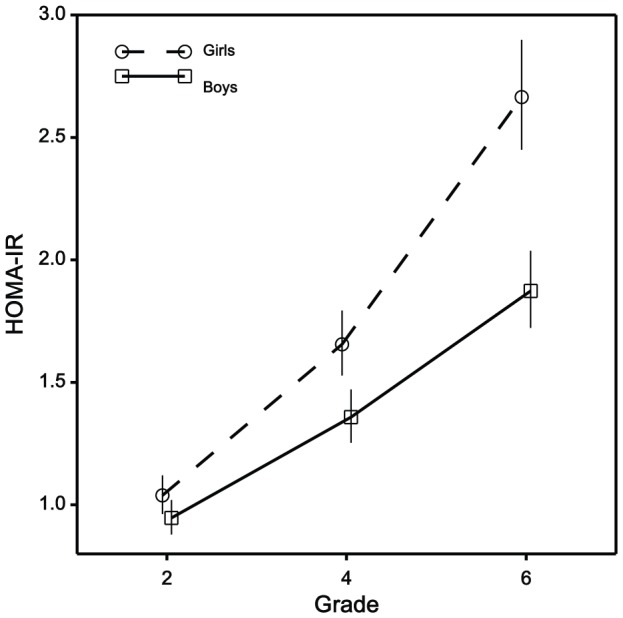
Mean values and 95% confidence intervals of HOMA-IR classified by sex and elementary school grades 2, 4, 6, where average age was 8, 10, and 12 years respectively.

Mean HOMA-IR levels increased progressively for both girls and boys (Figure 1). Corresponding medians (5^th^ and 95^th^ percentiles) for girls and boys respectively were: 1.04 (0.49–2.63) and 0.97 (0.37–2.10) at average age 8 years; 1.64 (0.64–4.04) and 1.27 (0.58–3.34) at age 10 years; 2.67 (1.20–6.45) and 1.79 (0.87–4.33) at age 12 years. These median HOMA-IR values were 9%, 27% and 49% greater in girls than boys at ages 8, 10, and 12 years respectively. By age 12 years, 23% of boys and 31% of girls had evidence of an elevated HOMA-IR of greater than 3, the suggested cutpoint for risk of metabolic syndrome [5].

As a further indication of the practical significance of the effect of changes in %BF and PA on IR in this age group, we provide an indication of the numbers of children whose IR was likely to be affected most by changes in %BF and PA between the ages of 10 and 12 years. Children benefitting most by a reduction in IR would be those who simultaneously reduced their %BF and increased their PA. Numbers (and percentages of the 12 year-olds) in this category with median, 5^th^ and 95^th^ percentiles of the changes are as follows. There were 61 females (24%) whose change in %BF was −1.9 (−6.7, −0.05) and change in PA Index 4.8 (0.17, 13.7); and 39 (14%) males whose change in %BF was −2.2 (−9.4,−0.2) and change in PA index 5.1 (0.47, −15.5). On the other hand children with increased %BF and reduced PA were those most likely to have increased their IR. There were 50 females (19.5%) in this category whose change in %BF was 2.0 (0.40, 6.6) and change in PA index −6.2 (−14.0,−0.58); and 87 males (31%) whose change in %BF was 1.94 (0.30, 7.3) and change in PA index −8.3 (−19.4,−0.90).

### Covariates of statistical significance

HOMA-IR was directly proportional to body weight for the boys (95% CI for proportionality constant 0.80–1.20) and girls (95% CI 0.90–1.30), and emerged as the strongest covariate of HOMA-IR (p<0.001); so body weight was included in the model when estimating individual relationships with other candidate explanatory variables (Table 2). The positive significant relationship between IR and body weight was sustained even after adjusting for %BF, indicating it reflected a characteristic other than adiposity, and this is discussed below. There were several other significant covariates of HOMA-IR, and for boys and girls data combined these were gender (p<0.001), grade (p<0.001), socioeconomic status (p = 0.02), the physical education intervention (p = 0.05), and pubertal rating (for the girls p = 0.002 in both grades 4 and 6; not significant for the boys in grade 4 (p = 0.3), but with a trend in grade 6, p = 0.065). However, when each of these variables was introduced into the model alongside body weight, which itself remained highly significant (p<0.001), the relationships between HOMA-IR and both PA and %BF were essentially unchanged.

**Table 2:** The longitudinal (within-child) relationships between HOMA-IR and explanatory variables percent body fat, physical activity, cardiorespiratory fitness and body weight.

	Boys		Girls	
	β (se)	p	β (se)	p
%BF	0.016 (0.008)	0.05	0.022 (0.009)	0.01
PA Index	−0.007 (0.003)	0.039	0.0005 (0.003)	0.85
Square root CRF	−0.06 (0.088)	0.51	−0.118 (0.094)	0.21
Log Wt	1.51 (0.219)	<0.001	1.646 (0.211)	<0.001

Adjustments were made for gender, school, socioeconomic status, pubertal rating and the logarithm of body weight, and relationships of IR with %BF were adjusted for PA index and vice versa. For the relationship with natural logarithm of body weight all other variables were ignored. The physical activity index is approximately the square root of average steps per day; CRF is cardiorespiratory fitness, the number of stages reached in the multistage run.

### Relationships with Insulin Resistance

We can express the relationships in Table 2 in more practical terms. These longitudinal (within-child) data indicate that if a girl reduced her %BF by 1 unit, her HOMA-IR was lowered by 2.2% (95% CI 0.04–4). Alternatively, an increase of %BF raised her HOMA-IR to the same extent. For the boys, a reduction in %BF by 1 unit lowered his HOMA-IR by 1.6% (95% CI 0–3.2); and an equivalent increase in HOMA-IR occurs as a result of a corresponding increase in %BF. As also illustrated in Table 2, longitudinal relationships between HOMA-IR and PA varied with gender. If a boy increased (or decreased) his PA by 2100 extra steps per day (10% change in the PA Index) his HOMA-IR was lowered (or increased respectively) by 3.5% (0.5–6.5), but there was no effect of PA on HOMA-IR in girls (p = 0.85).

This study focuses on longitudinal effects but it is of interest that cross-section relationships occurred between IR and %BF (p = 0.03 and 0.08 for boys and girls respectively), between IR and PA in the boys (p = 0.06) but not girls (p = 0.8), and also between IR and CRF (p = 0.04 and 0.03 for boys and girls). These cross-sectional relationships between IR and both %BF and PA were generally reflective of the longitudinal relationships, with the exception of the relationships between IR and CRF, where there was no evidence of any direct effects of CRF on IR at the longitudinal level.

### Classification of body composition

To assist a comparison of the body composition of this cohort with those of other studies, the children were classified according to their BMI. Approximately 24% of girls at the end of grade 2 at age 8 years were classified as overweight or obese, and in grade 6 at age 12 years, there were 23% girls in this combined category. For the boys, the respective percentages were 20% in grade 2 and 26% in grade 6.

## Discussion

In this prospective cohort study we provide new evidence that change in %BF has a direct (i.e. within-child) influence on the IR of preadolescent children as they approach 12 years of age; this being in contrast with the situation between the ages of 8 and 10 [3] where IR was not responsive to changes in %BF in either sex. On the other hand, the direct influence of PA on IR in the 8–10 year-old boys, but not girls, did persist over the next two years, reinforcing the gender specificity of this effect. Our data also show the direct effects of changes in %BF to be of practical as well as statistical significance; every 1 unit decrease (or increase) in %BF in a child brought about a reduction (or a respective elevation) in IR in the order of 2%. Considering this with the finding that from age 10 to 12, 19.5% of the girls and 31% of the boys had median increments of approximately 2%BF (with changes up to 7%BF), coinciding with considerable reductions in PA, the effects of commonly observed changes in %BF on IR are of sufficient size to be of practical significance. The importance of early attention to %BF and PA in preadolescence is further emphasized in that by age 12, 23 % of boys and 31% of girls had HOMA-IR in a range considered to put them at risk of metabolic syndrome.

Our analyses also revealed a finding which may apply to longitudinal studies in children in general. Body weight emerged as a significant explanatory variable for IR, independent of %BF, and failure to adjust for body weight may lead to different and potentially misleading evaluations of the relationships between IR and %BF or PA. Our study was not designed to determine why body weight emerged as a significant predictor of IR. However, in the interests of stimulating collegiate debate we suggest that body weight may represent influential factors associated with general growth and development. It is also noted that a significant relationship between IR and body weight might also occur should the DXA %BF measurement have a weight-related bias, but we have no reason to suspect this. The pertinent point is that adjusting for body weight permitted the calculation of more accurate relationships between IR and the variables of interest.

Our longitudinal data and objective methods of measuring %BF and PA show a progressive increase in IR over a 4-year period through to age 12 years, median HOMA-IR doubling in boys and increasing 250% in girls. It is sobering to report that at age 12 years, 23% of boys and 31% of girls in this apparently healthy cohort of Australian children had HOMA-IR values greater than 3, these levels considered to indicate risk of metabolic dysfunction [5]. However, the consequences of elevated HOMA-IR on the immediate and future health of children at this age is not well understood, and a better understanding of the degree of associated risk will only be gauged by continued longitudinal observation in cohorts such as this one.

We have previously reported the overall pattern of age-related changes in IR in describing reference ranges in healthy children [21]. We have also considered the age-related increases in IR previously demonstrated in our cohort as 8 to 10 year-olds [3] alongside another longitudinal study in England where IR actually decreased between 5 and 7 years of age [15], [22]. Our data suggested that any initial trends toward decreased IR are reversed by the age of 8, and we now report that IR continues to increase in both boys and girls through to age 12. The more pronounced increase in IR in girls compared with the boys from 10 to 12 years probably reflects the effect of relatively advanced pubertal development in girls [23].

Our study has both its strengths and limitations. Strengths of the study include the objective nature of the measurement of the key explanatory variables %BF and PA to accompany the accuracy of blood assays carried out in our hospital laboratory. However, objective measures by pedometers are limited in that they cannot measure some forms of PA, such as swimming and cycling; and pubertal development measurement was a limitation in that it was self-assessed. The reduction in precision of these measurements may have affected the estimation of some of the relationships. The statistical model and adjustment for effects of potentially confounding variables, including PA when investigating %BF and body weight when investigating all relationships was a solid aspect of the study; on the other hand a limitation was that we did not consider other potential covariates such as family history, diet, and sleep patterns. A major limitation however, is that the participants in this study were predominantly White children of mid-range socioeconomic status in a relatively affluent society. Consequently, our data may not be generally applicable to children of different ethnicity and living conditions.

In conclusion, the incidence of elevated IR even at age 12 years suggests that general community strategies to prevent elevated IR in pre-adolescents are worthy of consideration. Our longitudinal data indicate that strategies involving reduction in adiposity are likely to be successful because IR becomes directly responsive to changes in %BF in girls and boys between 10 and 12 years of age; but IR is also independently directly responsive to changes in PA in boys between the ages of 8 and 12. Finally we alert researchers to the importance of adjusting for body weight in longitudinal studies of IR in growing children because body weight is likely to emerge as a strong covariate independent of %BF, and failure to account for its effects may produce misleading outcomes.

## Supporting Information

Statistics S1 Statistical methods information [24].
(DOCX)
