# Supplementary material for: Effects of Changes in Adiposity and Physical Activity on Preadolescent Insulin Resistance: The Australian LOOK Longitudinal Study
Source: PLoS One. 2012 Oct 12;7(10):e47438. doi: 10.1371/journal.pone.0047438 (PMC3470575; doi:10.1371/journal.pone.0047438)
Supplement: Statistics S1 — Statistical methods information [24]. (DOCX) [file pone.0047438.s001.docx]

**Supporting Statistical Methods Information**

Our study design required that the model quantified relationships at three levels; between-child (within a school), within-child and between-school levels. Whilst the latter relationship is not directly relevant to the current investigation, it is important to account for it in determining relationships at the other two levels. Furthermore, the Lifestyle of our Kids (LOOK) study involved a variation of physical education as an intervention over successive years, and while the relationships of interest in the current study are not likely to be affected by a variation in school physical education, it was appropriate to include a term to adjust for any such effects. This was accomplished with the addition of the variable Group as set out below.

The response variables representing IR varied at the three levels as did the candidate explanatory variables PA, CRF weight and %BF. Other candidate explanatory variables such as our measure of socioeconomic status varied only at the school level. Our statistical model takes the form

HOMA-IR = constant + Group effect+ SES effect + PA_s_ + school random effect + Sex effect + Sex.Group interaction + PA_c_ + child random effect + Year effect + Sex.Year effect + Group.Year effect + PA_w_ + possible interactions between fixed effects + within-child random error;

where PA_s_ denotes the vector of PA means for each school; PA_c_ is the vector of differences between each child PA mean and the school mean; and PA_w_ is the vector of differences within each child, i.e. between repeat observations and the relevant child PA mean.

Similar models to the one above apply when PA is replaced by the other explanatory variables such as CRF or %BF. With PA as the example variable, as in the equation above, the current paper is concerned directly with PA_C_ and PA_w_, the relationships at the cross-sectional (between-child) and longitudinal (within-child) levels respectively. The above model fits within the general framework of general linear mixed models [[20](#_ENREF_20)]. Restricted maximum likelihood is used to estimate variance components and weighted least squares for estimating fixed effects. Statistical significance of effects was assessed by calculating adjusted Wald statistics [[24](#_ENREF_24)]. Explanatory variables were scaled by square root or logarithm to better meet linearity assumptions. General model checking procedures were routinely used to identify aberrant data and to check the model assumptions.
